# Successful renal transplantation to a recipient with type II cryoglobulinemia: a case report

**DOI:** 10.1186/s12882-018-0966-6

**Published:** 2018-07-09

**Authors:** Tomomichi Kasagi, Hironobu Nobata, Kaori Ikeda, Shogo Banno, Yasuhiko Ito

**Affiliations:** Division of Nephrology and Rheumatology, Department of Internal Medicine, Aichi Medical University School of Medicine, Nagakute, Aichi 480-1195 Japan

**Keywords:** Cryoglobulinemia, Renal transplantation, Double filtration plasmapheresis, Rituximab

## Abstract

**Background:**

Recurrence of glomerulonephritis is an important risk factor for renal graft dysfunction. Cryoglobulinemia is known as a relatively rare cause of renal failure, and doctors are usually hesitant to perform transplantation on a recipient with cryoglobulinemia because of the risk for graft loss. We present a case of renal transplantation on a patient with organ manifestations of type II cryoglobulinemia.

**Case presentation:**

At the age of 44 years, the patient developed acute kidney injury and purpura on the lower extremities with type II cryoglobulinemia after interferon therapy for hepatitis C virus. Cryoglobulinemic glomerulonephritis was suspected; however, despite immunosuppressive therapy combined with plasmapheresis, she eventually needed hemodialysis treatment. She was referred to us at the age of 49 years for renal transplantation. Cryocrit was 14% and the organ manifestations persisted, including the lower extremity purpura and neurologic symptoms. After monitoring and confirming sufficient suppression of cryoglobulin concentration by immunosuppressive treatment with prednisolone, cyclophosphamide, and rituximab combined with plasmapheresis, the operation was performed. After transplantation, the cryoglobulin concentration was continuously monitored, and plasmapheresis and rituximab infusion were performed as appropriate. Her graft function has remained stable for 2 years and 6 months.

**Conclusion:**

Our case suggested that a patient with cryoglobulinemia and persistent organ manifestations can receive a renal graft if the cryoglobulin concentration is sufficiently controlled by pretransplant treatment.

## Background

One important risk factor for renal graft dysfunction is de novo and recurrent glomerulonephritis, although rejection and infection are more common [1, 2]. Cryoglobulins are immunoglobulins that precipitate in a cold environment, can produce systemic organ damage [3, 4], and are known as a relatively rare cause of renal failure. Previous case reports have shown the recurrence of cryoglobulinemia in a renal allograft in patients who received a kidney transplant [3, 5, 6]. Those past experiences may cause doctors to hesitate to perform transplantation. We present a case of renal transplantation on a patient with organ manifestations of type II cryoglobulinemia. Good graft function was maintained for 2 years and 6 months after strict management of the cryoglobulin concentration by immunosuppressive therapy combined with plasmapheresis from the pretranplant period.

## Case presentation

**Fig. 1:**
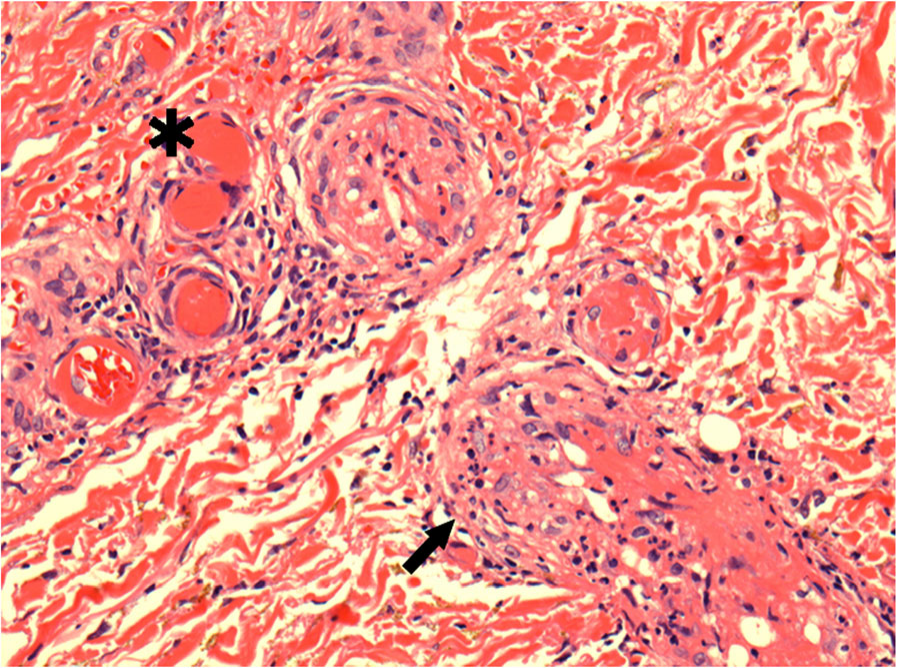
Vessel occlusions with hyaline thrombi (*) and inflammatory infiltrates (←) are seen (hematoxylin & eosin stain, × 400)

The patient was a 49-year-old woman who was diagnosed with hepatitis C virus (HCV) serotype 2 infection at the age of 29 years during pregnancy with her first child. She received interferon therapy, which afforded sustained virologic response. At the age of 41 years, she was diagnosed with macroglobulinemia based on a high serum IgM (2732 mg/dL) with M-protein of IgM-kappa by immunoelectrophoresis and the normal number of plasma cells in the bone marrow. She was asymptomatic and was followed-up without medication. At the age of 44 years, she developed acute kidney injury and purpura on the bilateral lower extremities with type II cryoglobulinemia, which was composed of monoclonal IgM and polyclonal IgG. Skin biopsy of the purpuric lesion revealed inflammatory infiltrates and small vessels with hyaline thrombi (Fig. 1).

Renal biopsy was avoided because of severe hypertension and thrombocytopenia, but cryoglobulinemic glomerulonephritis was strongly suspected. She received plasma exchange and immunosuppressive therapy with rituximab (RIT), cyclophosphamide (CPA), and glucocorticoid, but eventually needed hemodialysis treatment within the same year. The purpura of the extremities and neuropathy did not improve and she kept receiving double filtration plasmapheresis (DFPP) biweekly for cryoglobulin depletion. She requested living renal transplantation and was referred to us.

**Fig. 2:**
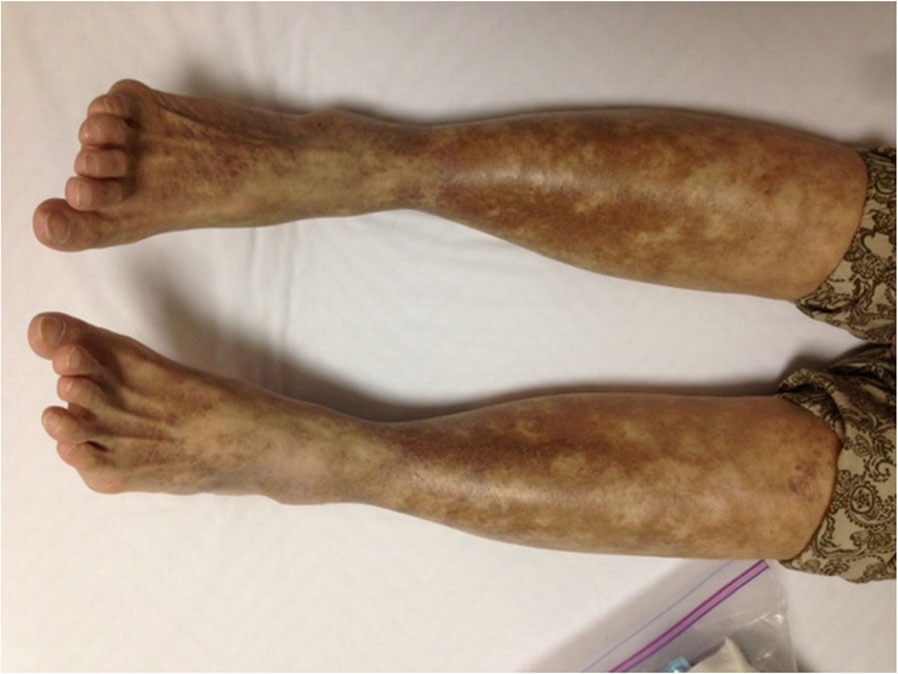
Livedo reticularis on the bilateral lower extremities

On our initial examination, livedo reticularis, hypothermoesthesia, and hypoalgesia on the bilateral lower extremities were observed (Fig. 2). Laboratory studies indicated white blood cell count 5300/μL, hemoglobin 10.6 g/L, platelet count 21.0 × 10^4^/μL, serum creatinine (Cr) 5.42 mg/dL, and C-reactive protein 0.47 mg/dL. IgG, IgA, and IgM were 1128.9 mg/dL, 211.8 mg/dL, and 371.1 mg/dL, respectively. Complement C3 was 79.0 mg/dL (normal range: 60–120 mg/dL), CH50 was 18.9 U/mL (normal range: 30–40 mg/dL), and rheumatoid factor (RF) was 2213.5 IU/mL. Although the recipient was negative for HCV-RNA on TaqMan quantitative assay, cryocrit was 14% and type II cryoglobulinemia was still demonstrated. As measured in our laboratory, the IgG and IgM concentrations within the cryoprecipitate (cryo-IgG and cryo-IgM) were 360.3 mg/dL and 261.3 mg/dL, respectively. An appropriate technique was necessary to get the correct value of cryoglobulin concentration, which can easily fluctuate and cause a reading error [7]. Blood samples were collected in pre-warmed syringes and tubes, clotted for 20 min, and centrifuged at 37 °C for 5 min; 1 mL of supernatant was collected and stored at 4 °C for 3 days. Then, the precipitate was dissolved in 1 mL of phosphate-buffered saline, and globulin concentration was measured. We routinely took an average of 2 or more measurements.

**Fig. 3:**
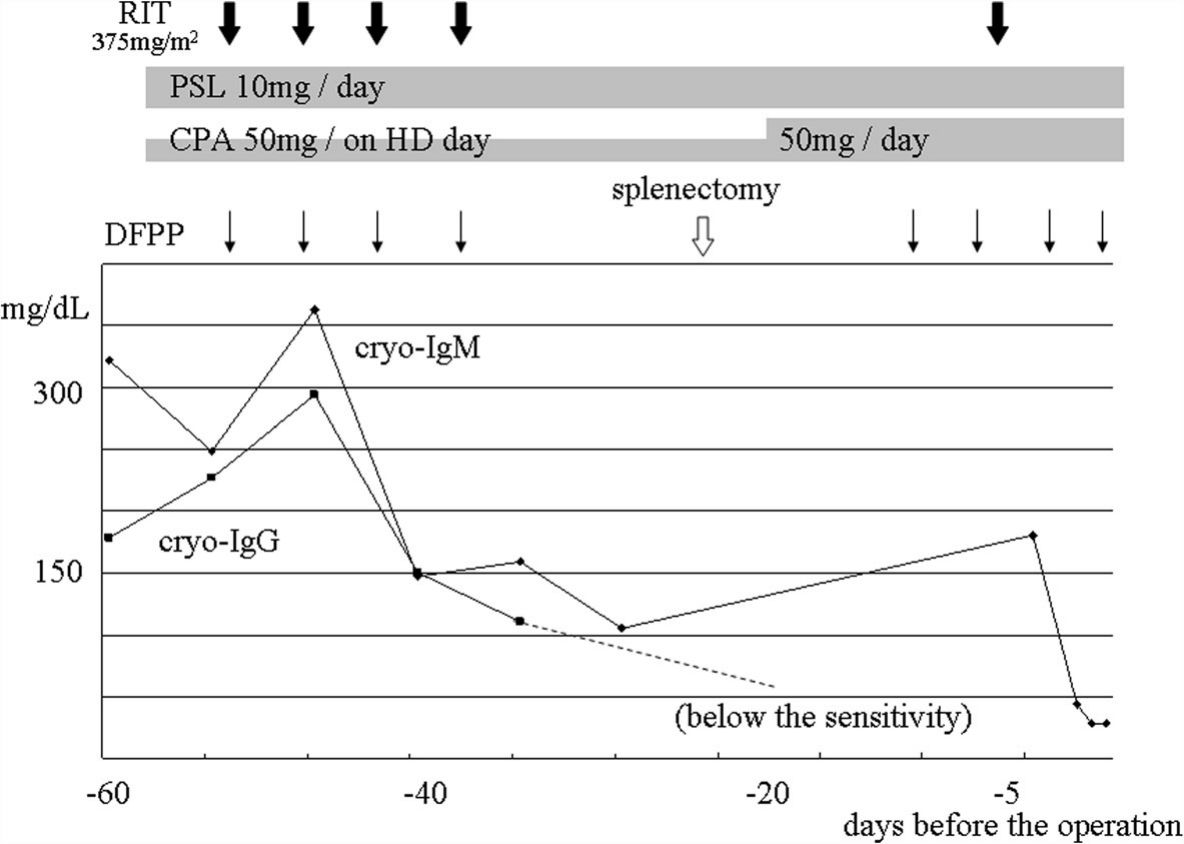
Pretransplant clinical course. RIT, rituximab; PSL, prednisolone; CPA, cyclophosphamide; HD, hemodialysis; DFPP, double filtration plasmapheresis

Because cryoprotein concentration was reported to correlate with the severity of symptoms and useful in monitoring response to treatment [4], living renal transplantation was planned when the concentration was suppressed enough by pretransplant treatment. The pretransplant clinical course is shown in Fig. 3. Prednisolone (PSL), CPA, and RIT were started 50 days before the transplantation, and DFPP and splenectomy were combined. Immediately before the operation, cryo-IgG and cryo-IgM were sufficiently suppressed and cryocrit was 0%.

The donor was her 70-year-old mother, whose left kidney was transplanted to the recipient’s right iliac fossa. During the operation, hypothermia was prevented by placing the recipient in a warm operating room and by giving warm fluid replacement and heating blankets.

**Fig. 4:**
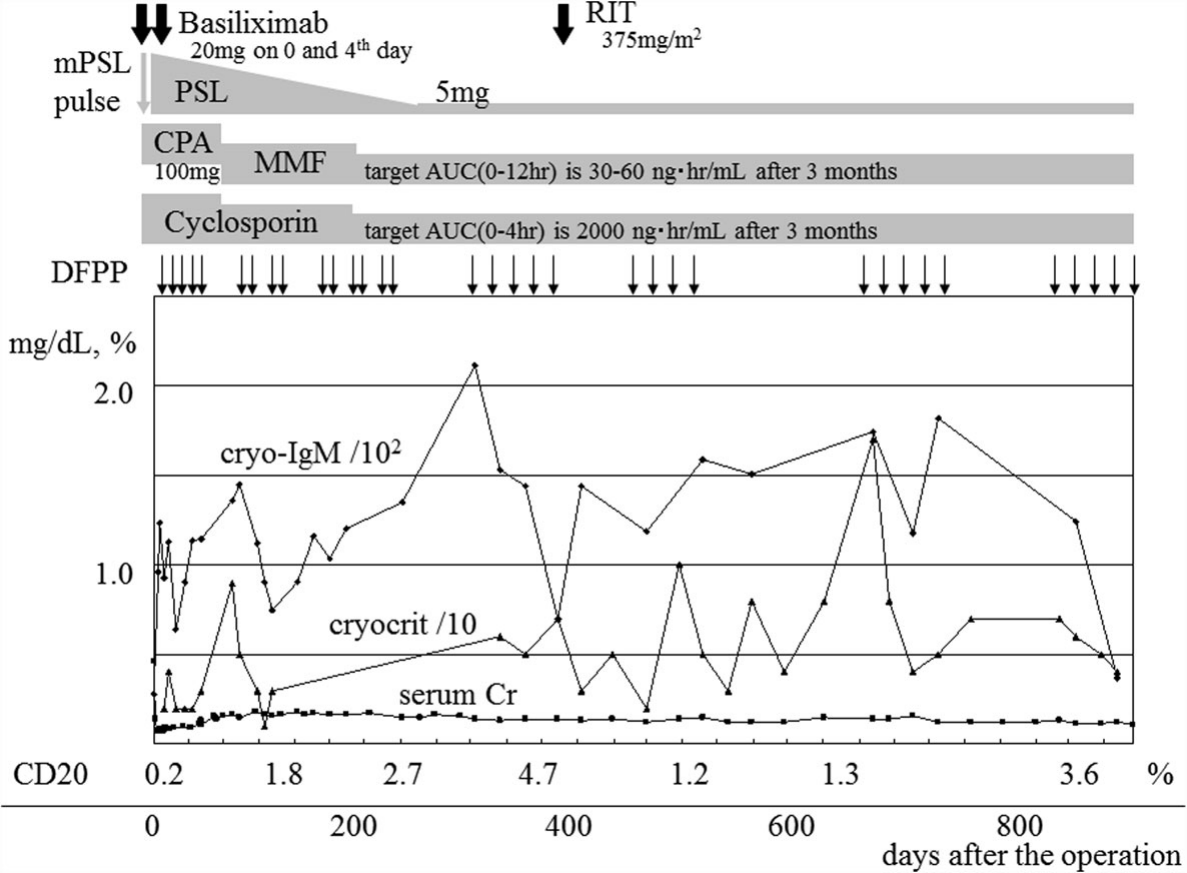
Posttransplant clinical course. AUC, area under the curve; CPA, cyclophosphamide; DFPP, double filtration plasmapheresis; MMF, mycophenolate mofetil; mPSL, methylprednisolone; PSL, prednisolone; RIT, rituximab

Basiliximab, PSL, CPA, and cyclosporin were used for posttransplant immunosuppression, and CPA was changed to mycophenolate mofetil 1 month after the operation (Fig. 4). The cryoglobulin concentration and the CD20-positive cell counts were monitored, and DFPP and RIT infusion were performed as appropriate. She remained stable with good graft function and improved purpura and neuropathy for 2 years and 6 months, without signs of recurrence.

## Discussion

Cryoglobulins are immunoglobulins that reversibly precipitate in a cold environment; 3 basic types are recognized according to the clonality [8]. Type II is predominantly associated with HCV, which interacts with the major extracellular loop of tetraspanin CD81 on lymphocytes and triggers chronic B cell stimulation [3, 9, 10]. Relapse of vasculitis despite sustained virological response had been reported in 1/3 of cases [11], suggesting that B cell proliferation can become independent of HCV over time. In our case, such B cell clones were suspected to exist.

Cryoglobulinemia is not rare in kidney transplant patients, particularly in patients with chronic HCV infection. Its prevalence was reported to be high, ranging from 37.8 to 81.2% [12–14], but in most cases, its concentration is relatively low. Moreover, the reported cryocrit was less than 1% in 92.9% of patients [12].

In our case, the risk of recurrence could not be ignored because of the high amount of cryoglobulins, with skin and neurologic involvement. There were few reports on renal transplantation in patients with cryoglobulinemic disease or with newly developed cryoglobulinemic disease after transplantation [5, 15–20]. In most cases, recurrence occurred at the early stage (i.e., within one year) after transplantation and graft loss could not be prevented once it developed (Table 1). Recently, some reports have shown the efficacy of RIT combined with plasmapheresis, but the cases in previous reports were asymptomatic and untreated before the recurrence. Our case was unique in that we attempted renal transplantation in a recipient with active manifestations of cryoglobulinemia but who was treated prophylactically. The overall outlook of cryoglobulinemia was reported to be poor in patients with high cryocrit, low C3 values, high creatinine at diagnosis, alveolar haemorrhage, or intestinal ischemia [3]. However, there is no study that determined the risk factors for recurrence after transplantation. Other reports showed that some signs, such as hypocomplementemia or elevated RF, can lead to recurrence [3, 19]. Those past experiences showed that the risk of transplantation was relatively high in cases with important organ complications, such as lung and intestinal involvement, low complement values, or poor control of cryocrit.

**Table 1:** Case reports of renal transplantions to patients with cryoglobulinemic disease or developing cryoglobulinemic disease after transplantation

year	numbers of cases	age	HCV	type	period to recurrence	results
1989	2		–	mixed	30 days	graft loss
			–	mixed	6 months	graft loss
1994	2	43	+	mixed	10 months	graft dysfunction (last serum Cr is not reported)
		52	–	mixed	5 months	graft loss
1996	1		+	TypeI MPGN	2 years	graft loss
2005	3	60	–	type 3	de novo	RIT prevented graft loss but died of infection
		56	+	type 3	de novo	RIT prevented graft loss (serum Cr is in 2 mg/dL)
		47	–	type 3	6 months	RIT prevented graft dysfunction (serum Cr is at baseline)
2006	7		+ in 5	mixed	de novo	RIT prevented graft loss in 5
			- in 2			2 died of infection
2010	1	50	–	type 2	4 years	graft loss
2013	1	46	–	type 2	35 days	GC prevented graft dysfunction (serum Cr is at baseline)

Our case report had some limitations. First, errors in the measurement of cryoglobulin can easily occur [7]. Although we paid enough attention and routinely took an average of 2 or more measurements, errors were still possible. Furthermore, other than our therapy adjustment, various factors may have contributed to the cryoglobulin concentrations and include general infection, use of other drugs, or influenza vaccination [3]. In our clinical course, the cryocrit values did not clearly respond to the treatment and were not perfectly correlated with the cry-IgM concentrations. Second, although high cryocrit values were reported to be an important risk factor for cryoglobulinemia recurrence [3], managing the cryocrit alone may not be enough. Third, graft biopsy was not performed because the patient was on anticoagulant to prevent cryoglobulinemia-related thrombus and the intestinal tract was located anterior to the transplanted kidney. Going forward, histological changes on biopsy samples need to be confirmed.

As of this writing, her graft function has remained stable for 2 years and 6 months. Our case suggested that a patient with cryoglobulinemia, even with the persistence of organ manifestations, can receive a renal graft when its concentration is sufficiently controlled by pretransplant treatment.

## Abbreviations

CPA: Cyclophosphamide
DFPP: Double filtration plasmapheresis
DSA: Donor-specific antibody
MMF: Mycophenolate mofetil
PSL: Prednisolone
RF: Rheumatoid factor
RIT: Rituximab
